# Metabolism fine-tunes macrophage activation

**DOI:** 10.7554/eLife.14354

**Published:** 2016-02-19

**Authors:** AnnMarie Torres, Liza Makowski, Kathryn E Wellen

**Affiliations:** 1 Department of Cancer Biology, University of Pennsylvania Perelman School of Medicine, Philadelphia, United States; 1 Department of Cancer Biology, University of Pennsylvania Perelman School of Medicine, Philadelphia, United Stateswellenk@exchange.upenn.edu; 2 Department of Nutrition, Gillings School of Global Public Health, University of North Carolina at Chapel Hill, Chapel Hill, United States

**Keywords:** macrophage metabolism, macrophage activation, immunometabolism, akt, acly, mTORC1, Mouse

## Abstract

A signaling pathway that rewires metabolism in macrophages to trigger changes in gene expression has been identified.

**Related research article** Covarrubias AJ, Aksoylar HI, Yu J, Snyder NW, Worth AJ, Iyer SS, Wang J, Ben-Sahra I, Byles V, Polynne-Stapornkul T, Espinosa EC, Laming D, Manning BD, Zhang Y, Blair IA, Horng T. 2016 Akt-mTORC1 signaling regulates Acly to integrate metabolic input to control of macrophage activation. *eLife* **5**:e11612. doi: 10.7554/eLife.11612

**Image** Macrophages are immune cells involved in several different processes (Credit: NIAID)

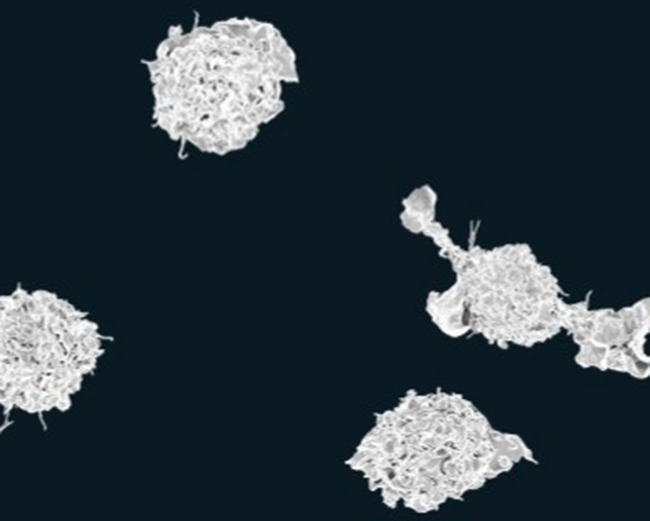

Cells continually monitor the availability of nutrients and alter their activities and metabolism accordingly. Immune cells are no exception (O’Neill and Pearce, 2015). Macrophages are important immune cells that perform many roles – they are, for example, involved in development and wound repair – but they need to be activated before they can carry out their functions. For years it was thought that there were two major subsets of activated macrophages: M1 macrophages that promote inflammation, and M2 macrophages that suppress the immune response. In reality, the distinction between the subsets is less clear, although it is thought that M1 and M2 macrophages have different metabolic profiles and phenotypes (O’Neill and Pearce, 2015). Nevertheless, it is poorly understood how metabolism contributes to the control of gene expression during the activation of immune cells, and how metabolism promotes the M2 phenotype in particular.

Now, in eLife, Tiffany Horng and colleagues – including Anthony Covarrubias as the first author – report on a signaling pathway that rewires the macrophage’s metabolism for the M2 phenotype (Covarrubias et al., 2016). First, they analyzed the metabolism of mouse macrophages that had been stimulated with a signaling molecule called interleukin 4 (IL-4) and confirmed several of the metabolic characteristics that were already known about M2 macrophages (e.g. that they show increased breakdown of fatty acids and oxidative phosphorylation in the mitochondria). However, the data also showed a characteristic that had previously been associated with the M1 phenotype (up-regulation of glycolysis). Covarrubias et al. went on to confirm that IL-4 triggered the uptake of glucose in a manner that depended on the kinase Akt. This kinase is well known as a key regulator of glucose uptake and metabolism (Pavlova and Thompson, 2016), and Covarrubias et al show that inhibiting Akt was shown to blunt the activation of some, but not all, M2 genes in response to IL-4.

These initial observations prompted Covarrubias et al. – who are based at Harvard, the University of Pennsylvania, Brigham and Women's Hospital, the Chinese Academy of Sciences, Drexel University and the University of Wisconsin-Madison – to investigate how the pathway that acts through IL-4 and Akt regulates specific M2 genes. Previously Akt activation had been shown to promote the phosphorylation of an enzyme called ATP-citrate lyase (or Acly for short). This enzyme converts citrate into acetyl-CoA, a substrate for other enzymes that acetylate histones. Thus, Akt can promote histone acetylation by regulating Acly (Lee et al., 2014). Acetylation of histones in turn causes the DNA nearby to become more loosely packed and allows the genes encoded within the DNA to be expressed. Covarrubias et al. found that stimulation by IL-4 led to increased acetyl-CoA production in the macrophages in a manner that depended on Akt and Acly. Importantly, they also found that IL-4 specifically induced histone acetylation at the promoters of those M2 genes whose expression depended on Akt. Furthermore, this acetylation was reduced if Akt or Acly were inhibited.

Covarrubias et al. also reported that macrophages that lacked a crucial subunit of a nutrient-sensing protein complex called mTORC1 expressed less Acly and activated certain M2 genes less strongly. Since mTORC1 activity is regulated by the supply of amino acids (Bar-Peled et al. 2014), Covarrubias et al. investigated if limiting this supply impaired the induction of the Akt- and Acly-responsive genes by IL-4. The answer was yes.

So, which genes responded to the signaling pathway that acted through IL-4, Akt, mTORC1 and Acly? Covarrubias et al. found that genes involved in the cell cycle and DNA replication, as well as chemokine genes, were regulated by both Akt and Acly in response to IL-4. Thus, the data suggest that Akt-mTORC1 signaling promotes Acly activity as a way to adjust specific responses, such as cell proliferation, depending on nutrient availability (Figure 1).

**Figure 1.**
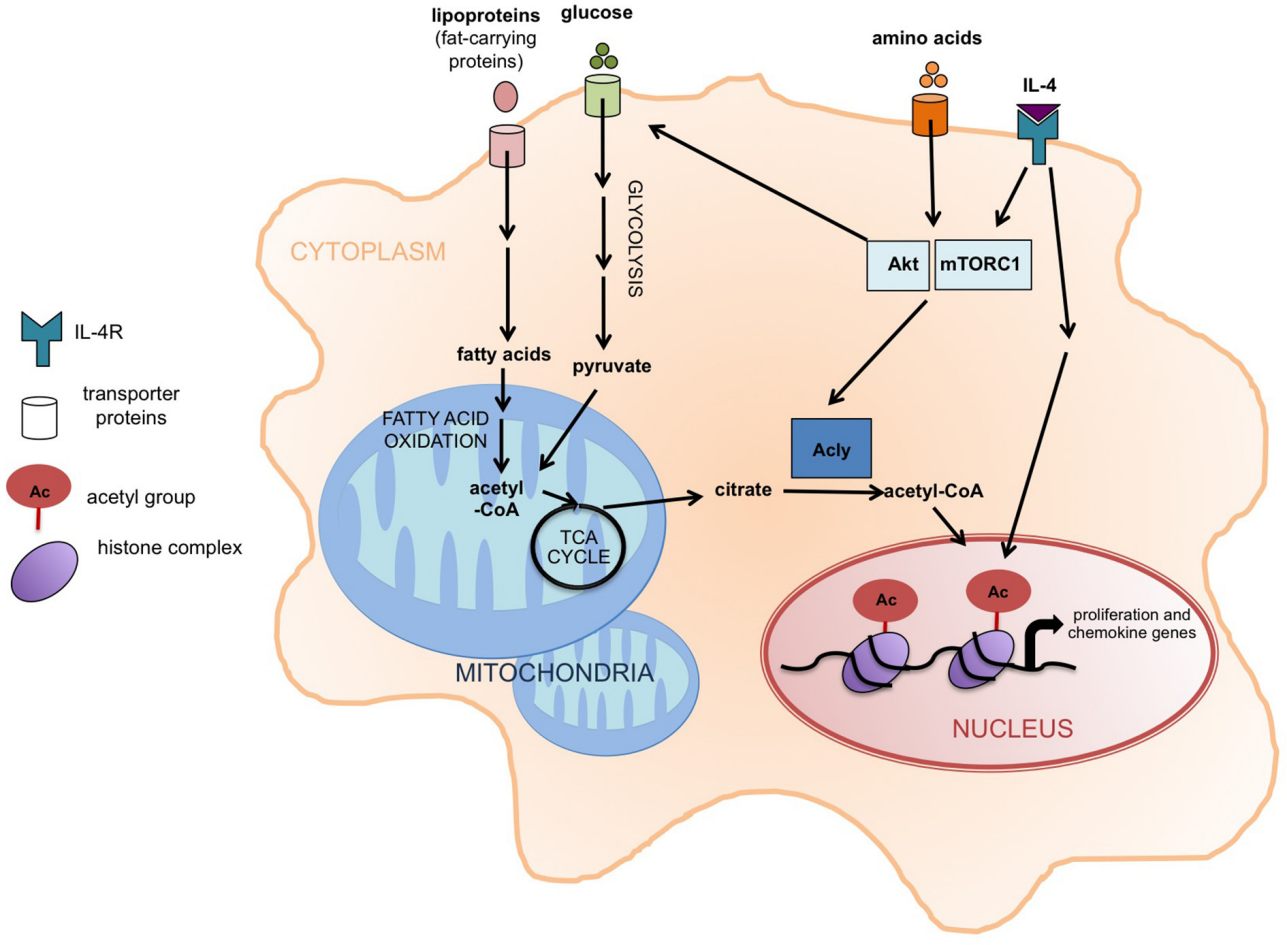
Akt-mTORC1 signaling and the activation of M2 macrophages. When the cytokine interleukin 4 (IL-4) stimulates its receptor (labeled IL-4R), it leads to the induction of at least two signaling pathways, including one involving Akt and mTORC1. Covarrubias et al. found that this pathway promotes the activation of an enzyme called Acly that converts citrate in the cytoplasm (shaded orange) into acetyl-CoA, which supplies the acetyl groups that are involved (via the modification of histones) in the expression of the genes that regulate the activation of M2 macrophages. These genes are involved in cell proliferation and chemokine production. The same pathway also promotes glucose uptake, glycolysis, and the production of acetyl-CoA. The increased levels of fatty acid oxidation in M2 macrophages also lead to more acetyl-CoA in mitochondria (shaded blue). It is possible that this acetyl-CoA is then converted to citrate via the TCA cycle and exported to the cytoplasm. Acly: ATP-citrate lyase;TCA cycle: tricarboxylic acid cycle.

It remains to be investigated how a metabolic enzyme such as Acly could regulate the expression of specific gene sets. However, recent studies have identified other acetyl-CoA-producing enzymes in complexes with histone modifiers and DNA-binding proteins (Matsuda et al., 2015; Li et al., 2015). These findings suggested that the enzymes might provide a source of acetyl-CoA for histone acetylation that is localized to specific genes. It is possible that Acly is recruited to specific subsets of M2 genes through similar interactions.

Glucose is the major carbon source for histone acetylation in other cell types (Evertts et al., 2013), but it is possible that different nutrients (e.g. fatty acids) could impact histone acetylation in M2 macrophages. The breakdown of fatty acids by oxidation (as occurs in M2 macrophages) generates acetyl-CoA in mitochondria. Through citrate export and the activity of Acly, this might lead to more acetyl-CoA in the cytoplasm or nucleus, which could then be used to modify gene expression (Figure 1). Since the Acly enzyme is typically activated when fatty acids need to be synthesized (rather than oxidized), it is tempting to speculate that Acly might be activated in M2 macrophages primarily to promote histone acetylation at nutrient-responsive genes.

Another key question is how metabolic fine-tuning of macrophage activation contributes to their activity in vivo. M2 macrophages promote sensitivity to insulin and maintain metabolic health, but they can also support tumor growth. In diseases such as diabetes or cancer, macrophages that are present in obese fat tissue or in tumors often have limited access to oxygen and nutrients, such as glucose, due to poor blood supply (Pavlova and Thompson, 2016; Sun et al. 2011). Recently, glucose restriction within tumors was shown to impair the anti-cancer activity of other immune cells called T cells (Sukumar et al., 2015). Similarly, understanding how metabolic conditions within fat tissue or tumors impact the IL-4-Akt-mTORC1-Acly signaling axis could provide new insights into the roles of macrophages in metabolic health and tumor progression. Metabolic context thus plays an important role in determining the activity of macrophages.
